# Prosociality is centred on intentions, not outcomes

**DOI:** 10.1007/s10071-025-01981-y

**Published:** 2025-07-31

**Authors:** Leon Li, Tindaya Déniz, Louisa Huff, Solveig Jurkat, Manuela Missana, Laura Tietz, Sebastian Grueneisen

**Affiliations:** Faculty of Education, Leipzig University, Marschnerstr. 29a, 04109 Leipzig, Germany

## Abstract

A recent proposal defines prosociality in terms of intentional behaviour that successfully benefits another. We suggest, however, that prosociality would be better defined more simply in terms of the intention to benefit another, regardless of whether a benefit really occurs, for three reasons: (i) the construct of “benefit” is itself difficult to operationalize, (ii) an intention-based definition is no less conservative than the proposed outcome-based one, and (iii) an outcome-based definition overlooks plausible cases of intended but unsuccessful prosocial actions as well as the psychological consequences of such actions. Amending the working definition of prosociality to be centred on intentions, not outcomes, would make it more useful as a tool for comparative and developmental research.

In a recent contribution to *Animal Cognition*, Kopp et al. (2024) propose an ambitious conceptual framework for comparative research on prosociality. We commend their proposal for its theoretical precision and expansive scope. A remarkably rich catalogue of different kinds of behaviours from numerous animal species, ranging from food sharing to alloparenting to group defence, are integrated by Kopp and colleagues under one operational definition of prosocial behaviour. Specifically, Kopp et al. (2024) define prosocial behaviour as “voluntary, intentional behaviour that results in benefits for another” (p. 3) based on Eisenberg and Miller (1987). As the authors note, this definition “is narrower than other definitions of prosociality.. as it does not include intentional, but unsuccessful attempts to benefit another. This conservative feature has the advantage that it relies on observable behaviours with a defined outcome. It does not require researchers to assume actors’ intended goals when actors fail to achieve those goals with their action(s)” (Kopp et al. 2024, p. 3).

Respectfully, in our view, to be adopted as a working definition of prosociality for comparative and developmental research, Kopp and colleagues’ proposal would benefit from an amendment. Our three main critiques are as follows. Firstly, their focus on benefits does not simplify the operationalization of prosociality because the construct of “benefit” is itself difficult to operationalize. Secondly, an alternative and simpler definition of prosociality focusing on intentions, not benefits, is no less conservative than their original proposal. Thirdly, their requirement that an action must *succeed* in benefitting another overlooks plausible cases of intended but unsuccessful prosocial actions as well as the psychological consequences of such actions.

But how should prosociality be defined, then, if not according to their proposal? We suggest that a simpler definition focusing only on intentions, not outcomes, would be suitable, such as this one from Eisenberg et al. (1999): “voluntary behavior intended to benefit another” (p. 1360). We acknowledge that prosocial behaviour has been defined in various ways across disciplines, from acts that transfer resources to acts that elicit societal approval (Jensen 2016; Pfattheicher et al. 2022), and we would not claim that our preferred definition is the only relevant one for all contexts. Yet, for comparative and developmental research, a definition based on intentions has proven exceptionally useful and is likely to remain a productive framework for future research. To be clear, we do not equate prosociality with altruism. As Eisenberg and Miller (1987) point out, prosocial behaviours may stem from a variety of motives, whether altruistic (benefitting a recipient out of genuine concern for the recipient) or selfish (benefitting a recipient as a means of obtaining a benefit for oneself). In line with Kopp et al., we focus on prosociality in the broader sense rather than altruism specifically. We return to this issue in the future directions, where we discuss how an intention-based definition is an appropriate framework for further research on the underlying motives of prosocial actions.

## Ambiguities in defining benefit

To begin, the requirement that a “benefit” must occur for an action to count as prosocial is not without its own ambiguities. On the contrary, it raises a number of additional open questions as to how “benefit” should be operationalized:

1. Whose perspective is the basis for assessing whether an action was beneficial or not? Is it the *recipient’s* perspective? If so, then paternalistic helping, in which the kind of help given by an actor differs from what the recipient herself wants (i.e., is viewed by the recipient as not beneficial) would be classified as not prosocial, a conclusion that seems intuitively wrong. Surely paternalistic helping – such as a parent putting on a child’s seatbelt against the child’s disapproval – is prosocial, even if a recipient does not appreciate it (Martin et al. 2016). Or is it the *actor’s* perspective? If so, then the definition of prosocial behaviour is reducible to a simpler intention-based definition: An action is prosocial if the actor thinks it will benefit the recipient, regardless of whether a benefit really occurs. A third option is that the *researcher* determines whether an action was beneficial based on some objective standard of helpfulness (e.g., improving a recipient’s emotional or physical well-being). But this is incredibly difficult to operationalize on practical, theoretical, and moral grounds (e.g., giving someone an unhealthy snack alleviates their hunger but weakens their long-term health).
2. Do staged “benefits” count as prosocial? In numerous demonstrations that human toddlers and other animals act prosocially, researchers have used experimental settings that were artificially staged to give actors the opportunity to “help” (e.g., by intentionally dropping an object out of reach, thereby giving an actor the opportunity to retrieve it; Warneken and Tomasello 2009). In such cases, no real benefit occurs, as the “problem” that the actor helps solve was not a real problem to begin with. Staging opportunities for children to “help” with tasks, although doing so may be more time-consuming and inconvenient than simply doing the tasks oneself, is also common in Western socialization practices (Hammond and Carpendale 2015). In our view, such acts still warrant being considered prosocial, despite the artificiality of their benefits, based on the fact that the actors themselves think they are benefitting the recipients. Allowing such acts to be defined as prosocial would also help ensure the utility of the definition of prosociality as a tool for comparative and developmental research.
3. What is the timeframe for assessing whether a benefit has occurred? The benefits of some actions are not realized until some time after they are completed. Would such actions be considered “not prosocial” until and unless a benefit is eventually realized? Suppose, for instance, that an actor gives a partner a tool to obtain a reward. If the partner loses the tool on the way to the reward, then no benefit will have occurred. Or suppose that an actor shares food with a partner that turns out to be contaminated. The action seems beneficial in the moment but is detrimental down the line. Yet, we would argue that such acts of tool and food sharing should still count as prosocial. What matters is simply that the actor intends to benefit the recipient, regardless of whether a benefit really occurs. Additionally, some actions may generate no immediate benefits but instead pave the way for future benefits (e.g., teaching one’s offspring how to acquire food rather than giving the food directly may take time and delay the offspring’s feeding, but it prepares the offspring to become independent in the future).

## An intention-based definition is also conservative

Another argument in favour of an intention-based definition is that it is actually no less conservative than Kopp et al.’s proposal. To illustrate our point, Table 1 depicts a factorial crossing of two dimensions by which actions may be described: Intention (Prosocial intention vs. No prosocial intention) and Outcome (Beneficial outcome to a partner vs. No beneficial outcome to a partner).

**Table 1:** Four categories of actions

	**Prosocial intention**	**No prosocial intention**
**Beneficial outcome to a partner**	Successful intentional benefitting	Accidental benefitting
**No beneficial outcome to a partner**	Unsuccessful attempted benefitting	Not benefitting

Kopp and colleagues’ definition is not solely outcome-based. That is, even when it is given that an action has resulted in a beneficial outcome to a partner, Kopp and colleagues do not necessarily therefore assume that the action was prosocial. Instead, they are willing to assess whether the action was intentional or not: “our definition requires individuals to act intentionally.. we will not consider behaviours as prosocial actions that accidentally provide benefits to others” (Kopp et al. 2024, p. 3). We completely agree that prosocial “successful intentional benefitting” may be distinguished from non-prosocial “accidental benefitting” on the basis of their different intentions, even if their outcomes are identical.

What we would further assert is that prosocial “unsuccessful attempted benefitting” and non-prosocial “not benefitting” may also be distinguished in this way—on the basis of their different intentions, even if their outcomes are identical. The reasoning is the same in both cases: Actions that have the same value on the Outcome dimension (i.e., belong to the same horizontal row in the table) may nonetheless differ on the Intention dimension (i.e., belong to different vertical columns in the table). Kopp and colleagues apply this reasoning to differentiate between cells in the top row of the table. It is arguably not any less conservative to apply this reasoning to the bottom row as well.

## Advantages of an intention-based definition

Our third objection to Kopp et al.’s original formulation is that it is overly restrictive—it precludes researchers from tapping into rich sources of data on both the actors who attempt, at times unsuccessfully, to act prosocially as well as the actors’ social partners, who evaluate and respond to the unsuccessful attempts. To the first point, at least within psychology, we are interested in the cognitive mechanisms that underlie behaviours, independent of whether the behaviours fully achieve their intended results. An intention-based definition allows us to take a developmental focus on early-emerging precursors of behaviours in yet-to-be-fully-competent children, whereas such data would be discounted by an outcome-based definition that regards only “successful” actions as valid data. Analogously, consider how constrained research on language development would be if it focused only on “successful” speech acts and discounted data on “unsuccessful” precursors of linguistic competence such as children’s babbling and speech errors.

To the second point, prosocial behaviours, even if unsuccessful, can have significant social consequences. Humans judge and respond to others’ actions based not only on their outcomes but also their intentions (Young and Saxe 2011; Zelazo et al. 1996). Indeed, even infants and apes distinguish between individuals who are *unwilling* versus *unable* to help (Behne et al. 2005; Call et al. 2004). For instance, infants preferentially interact with unsuccessful helpers over successful hinderers (Hamlin 2013) and preferentially help experimenters who are unable versus unwilling to share with them (Dunfield and Kuhlmeier 2010). Focusing only on successful prosocial acts might thus overlook important aspects of the social dynamics that are triggered by unsuccessful prosocial acts (e.g., with regard to reciprocity, social bonding, reputation formation, and signalling of cooperative motives).

## Hypothetical examples

In two tables, we give further examples of actions that challenge the requirement that a benefit must occur for an action to count as prosocial. The examples strike us as being clearly prosocial, despite not delivering real benefits to recipients. To begin, Table 2 catalogues behaviours observed in human toddlers.

**Table 2:** Non-beneficial prosocial actions in toddlers

Food sharing	A toddler offers food to a partner, but the food is undesirable (e.g., picked up from the ground or allergenic to the partner).
Comforting	A toddler tries to console a distressed partner through ineffective means (e.g., sharing toys with an adult), and the partner remains distressed.
Instrumental helping	A toddler assists with a task, but the clumsy assistance slows down the task’s completion.
Object retrieval	A toddler tries to retrieve an object, but the object is inaccessible to the toddler.
Informing	A toddler tells a partner who is searching for an object the (previous) location of the object, not knowing that the object is no longer there.

Cases of non-beneficial food sharing, comforting, and instrumental helping could plausibly also occur in non-human animals, such as primates. Table 3 catalogues additional examples of prosocial behaviours that could plausibly be observed in primates. Altogether, the examples may help illustrate the richness and range of prosocially motivated behaviours that would be discounted as not prosocial according to a definition centring outcomes.

**Table 3:** Non-beneficial prosocial actions in primates

Territory defence	A primate confronts an invading rival group or predator but gets injured and fails to deter the invader.
Conflict resolution	A primate intervenes between two conspecifics in a fight, but they continue fighting.
Infant transport	A primate forms a bridge between trees with her body to let her infant cross, but the infant falls off.
Tool provision	A primate gives a tool to a conspecific, but the conspecific does not use it or attempts to use it but fails.
Granting passage	A primate opens a door for a conspecific, but the conspecific does not go through it.

## Future directions

A focus on the intentions behind prosocial actions invites exciting avenues for future research. For instance, one ever-present challenge for psychological research on intentions is to pinpoint the specific *motive* behind an action. Even if it is clear that an animal acted intentionally, it may still be unclear what the motive behind the action was. In the case of prosocial actions, possible motives include not just the altruistic motivation to benefit the recipient but also selfish motivations, such as gaining status or eliciting reciprocity (Jensen 2016; Wilson 2006). Thus, a key goal for future research is to employ experimental designs that afford targeted interpretations of the specific motives behind prosocial actions.

A recent review of research in this direction with human children was provided by Grueneisen and Warneken (2022). As described in their review, children’s early prosocial actions appear to be motivated by a genuine concern for the recipient, as such actions are *not* experimentally boosted by “material or reputational incentives” (p. 324). With age, children additionally acquire a more strategic kind of prosociality, benefitting others as a “means to achieve ulterior goals such as to improve their reputation, to be chosen as social partners, to elicit reciprocity, and to navigate interpersonal obligations” (p. 323). In another review of useful examples of experimental research on motives, Engelmann and Rapp (2018) presented evidence that children act more prosocially when observed by others, an indication that one of the motives underlying children’s prosocial behaviour is the desire to form a positive reputation. A recent study by Giner Torréns et al. (2021) suggests that, for 18-month-old toddlers, an interest in social interactions may be a driving force behind prosocial behaviours. Further research into the motives behind prosocial behaviours in nonhuman animals would be highly valuable.

Another avenue for future investigation is the evolution of prosociality, a line of inquiry that requires careful attention to the distinction between proximate and ultimate levels of analysis. The proximate level pertains to questions about psychological states, such as whether an animal intended to benefit a recipient or what motive drove its action. The ultimate level pertains to questions about evolutionary fitness, such as how a behaviour impacts the fitness of the actor and others. The two levels are distinct, as noted by Jensen (2016): “actions can be altruistic at one level but not another. For example, an actor can be selfishly motivated, as in strategic reciprocity, yet improve the fitness of others, or the actor might reduce the fitness of others despite intending to help them” (p. R749).

From an evolutionary perspective, prosocial behaviours, especially costly ones, may appear to pose a conundrum: How could behaviours that apparently reduce an animal’s own fitness in favour of another’s have evolved? In response to this conundrum, evolutionary accounts of prosocial behaviour often appeal to mechanisms such as kin selection, group selection, or reciprocal altruism (Jensen 2016; Wilson 2006). Testing these hypotheses requires further experimental research, along with careful consideration of the fitness consequences of prosocial behaviours in their natural contexts. When it comes to these kinds of evolutionary analyses about the ultimate functions of behaviours, what matters is indeed the actual fitness *outcomes* of the behaviours, not the behaviours’ underlying intentions, as natural selection only operates on what actually occurs, not on what individuals may have intended to occur (Okasha 2020). Thus, we agree with Kopp et al. that the actual outcomes of behaviours are highly important data to consider—but, in our view, more so for evolutionary questions about ultimate functions than for psychological questions about proximate mechanisms and their ontogeny.

## Conclusion

In closing, we assert that prosociality is a construct that is best defined in terms of intentions, not outcomes. This also reflects the psychological reality of how perceptions of others’ acts can be based on intentions, in addition to outcomes. Requiring realized benefits to others as part of the definition of prosociality restricts the utility of the definition for comparative research on a wide array of behaviours whose benefits may not always be clear, immediate, or successful. We hope that our suggested amendment to Kopp et al.’s definition will help facilitate future investigations into diverse forms of prosocial behaviour across species (successful or otherwise).

## Data Availability

No datasets were generated or analysed during the current study.
